# *Enterococcus casseliflavus* Bacteraemia in a Patient with Chronic Renal Disease

**DOI:** 10.3390/idr12030015

**Published:** 2020-11-04

**Authors:** Alexandra Vasilakopoulou, Sophia Vourli, Nikolaos Siafakas, Dimitra Kavatha, Nikolaos Tziolos, Spyros Pournaras

**Affiliations:** 1 Laboratory of Clinical Microbiology, Medical School, Attikon University General Hospital, National and Kapodistrian University of Athens, 124 62 Athens, Greece; svourli@med.uoa.gr (S.V.); nsiaf@med.uoa.gr (N.S.); spournaras@med.uoa.gr (S.P.); 2 4th Department of Internal Medicine, Medical School, Attikon University General Hospital, National and Kapodistrian University of Athens, 124 62 Athens, Greece; dimitra.kavatha@gmail.com (D.K.); r-nikolaos.tziolos@hotmail.com (N.T.)

**Keywords:** *Enterococcus casseliflavus*, bacteraemia, thrombophlebitis

## Abstract

*Enterococcus casseliflavus* is a rare pathogen that usually causes urinary tract and abdominal infections. Its main characteristics are positive motility, yellow colonies and constitutive low-level resistance to vancomycin. We present a case of *E. casseliflavus* bacteraemia due to thrombophlebitis at the site of the central venous catheter used for hemodialysis in a renal patient. The biochemical identification of the microorganism was further corroborated by molecular detection of the *vanC* gene. The patient received antibiotic therapy initially with daptomycin and gentamicin, and then with ampicillin and ceftriaxone. The outcome was cure, and he was released from the hospital after seven weeks afebrile with negative blood cultures.

## 1. Introduction

*Enterococcus casseliflavus* is a member of the normal stool flora and has constitutive low-level resistance to vancomycin. It is rarely isolated from clinical specimens but may cause serious invasive infections [1,2,3]. An unusual case of septic thrombophlebitis and bacteraemia by *E. casseliflavus* is presented. 

## 2. Case Report

A 72-year-old man was referred to our hospital from a suburban nephrology center because of prolonged fever and persistent enterococcal bacteremia. The patient was suffering from chronic renal failure, undergoing regular hemodialysis sessions three times per week through a permanent right internal jugular venous catheter. He was a chronic smoker with chronic obstructive pulmonary disease, and had been operated on for lung decortication because of amiantosis 18 years prior to admission (since then he was under methylprednisolone therapy). Furthermore, he had high blood pressure and single vessel coronary artery disease for which he was treated with successful angioplasty the previous year, and was compliant to antihypertension and anticoagulant therapy. 

The patient had sought medical consultation 35 days before in the nephrology center where he was having hemodialysis, because he had high fever (up to 39 °C). Blood cultures were drawn and were processed in the nephrology center’s laboratory, resulting in the isolation of *Enterococcus spp.* from two culture vials with a susceptibility profile that included vancomycin: sensitive (MIC: 4 μg/mL), ampicillin: sensitive, daptomycin: sensitive, dalfopristin/quinopristin: resistant, gentamicin synergy: sensitive, ciprofloxacin: sensitive, and linezolid: sensitive. The patient received daptomycin and ciprofloxacin for 21 days. After the completion of antibiotic therapy he presented again with fever at the nephrology center, new blood cultures were drawn, and he started again receiving daptomycin. Two days later, during hemodialysis, he suffered from chills, and after completion of the session developed fever. In the next hemodialysis session, the same symptoms occurred (chills and fever), and gentamicin was added to his therapy. The nephrology clinic’s laboratory in the meantime isolated again *Enterococcus spp.* from the blood culture. He was treated with antibiotic lock therapy of the central venous catheter with gentamicin, and was referred to University General Hospital Attikon for further evaluation and treatment. 

On the day of admission to our hospital, the following laboratory results were obtained: hemoglobin 10.4 g/dL, leukocytes 13,590/μL (Neutrophils 87%), platelets 295,000/μL, creatinine 3.8 mg/dL, C-reactive protein 61 mg/L, and procalcitonine 2.7 ng/mL. The physical examination was normal except for a holosystolic mitral valve murmur upon cardiac auscultation. Initially he continued the therapeutic regimen he had started at the nephrology center (antibiotics only on the haemodialysis days of Tuesday, Thursday and Saturday). On Tuesday and Thursday, he was receiving daptomycin 300 mg IV, and on Saturday the dose was 450 mg. On all haemodialysis days he was receiving gentamicin 240 mg IV before the haemodialysis session and gentamicin 80 mg IV after each session. On the first day at the hospital six blood cultures were drawn and two more on the following day, and all were incubated with the standard BACTEC system (Becton Dickinson, Sparks, NV, USA). On the second day of his hospital stay, five of the blood cultures were positive with Gram-positive coccus forming chains. On the third day, two more cultures were positive with the same coccus. The central venous catheter was removed on the third day of stay. The isolate from all seven blood culture bottles was identified as *E. casseliflavus*. The colonies in standard Columbia agar exhibited distinct yellow pigment, the coccus was positive for motility [4], and the biochemical profile examined by Phoenix (Becton Dickinson, Sparks, NY, USA) and Vitek 2 (BioMerieux, Marcy-l’Etoile, France) matched *E. casseliflavus*. The antibiotic susceptibility phenotype included vancomycin: sensitive (MIC: 4 μg/mL), ampicillin: sensitive (MIC: 1 μg/mL), daptomycin: sensitive (MIC: 2 μg/mL), dalfopristin/quinopristin: resistant (MIC: 8 μg/mL), gentamicin synergy: sensitive (MIC: 16 μg/mL), ciprofloxacin: sensitive (MIC: 1 μg/mL), and linezolid: sensitive (MIC: 4 μg/mL). The MICs were determined by Vitek 2, Phoenix and E-tests while following the EUCAST breakpoints for all antibiotics (with the sole exception of the daptomycin MIC which was compared to the CLSI breakpoint). Furthermore, a multiplex PCR detecting *vanA, vanB, vanC, vanD, vanE* and *VanG* genes was performed and was positive for *vanC*, further corroborating the biochemical diagnosis [5]. The central venous catheter was cultured but there was no growth of any pathogen. The urine cultures were also negative.

The transesophageal echocardiogram was negative for vegetations and the fundoscopy was negative for Roth spots in the retina. A colonoscopy revealed numerous diverticula mainly in the sigmoid colon. CT scans of the head, thorax and abdomen were performed revealing a thrombus in the anterior vena cava near the distal end of the central venous catheter, and a pulmonary embolus at the lower lobe of the right lung involving the respective lobar artery and segmental branches. 

After the fourth day in our hospital the patient’s antibiotic therapy was changed to a combination of ampicillin 2gr IV every 8 h and ceftriaxone 2gr IV every 12 h, which he received for a total of 6 weeks. He also received anticoagulant therapy for the pulmonary embolus. He was released from the hospital after a total stay of seven weeks, afebrile, with negative blood cultures. 

## 3. Discussion and Conclusions

*E. casseliflavus* is a yellow motile enterococcus that can cause serious infections mainly in immunocompromised and chronically ill patients [6]. Our patient was particularly vulnerable because he was under methylprednisolone therapy and suffered from renal failure, coronary artery disease and chronic obstructive pulmonary disease. He carried a permanent central venous catheter that caused thromobophlebitis at the anterior vena cava, and since the patient suffered from fever and chills after the hemodialysis sessions it is presumed that he had septic thrombophlebitis.

The imaging findings in combination with the positive blood cultures suggested enterococcal thrombophlebitis and probable consequent septic pulmonary embolism. Since *E. casseliflavus* was not isolated from any other culture, we can only assume the primary source of infection. The three most probable sources would be the urinary tract, the central venous catheter and the gastrointestinal tract. The urine cultures where negative, thus excluding the urinary source. The culture of the catheter was negative, but this may be attributed to the antibiotic lock therapy. Another possible course of events would include, first, thrombophlebitis due to mechanical irritation by the catheter, and then colonization of the thrombus and the surrounding tissues by *Enterococcus* circulating in blood. Because the patient had numerous diverticula in the sigmoid, these could have served as the source of transient enterococcal bacteraemia leading to thrombus colonization. 

The initial two isolates from the positive blood cultures in the suburban nephrology center, which were identified there as *Enterococcus spp.*, had identical susceptibility profiles to the *E. casseliflavus* isolated in our hospital’s laboratory, suggesting the same strain caused all bacteraemic episodes. 

The identification as *E. casseliflavus* in the university hospital was based on the biochemical profile of the microorganism, the detection of the *vanC* gene, the positive motility and the yellow pigment. The combination of all the above traits was conclusive for *E. casseliflavus* [7,8]. Previous studies have proven the importance of the last two properties for correct identification at the species level since *E. casseliflavus* is the only motile *Enterococcus* producing yellow pigment [9]. The yellow pigment of the colonies was more evident after 48 h of incubation, and by inspecting the color on a white cotton swab. The antibiotic susceptibility profile of the isolate, with a relatively high MIC for vancomycin and low MICs for teicoplanin and ampicillin, is also indicative of *E. casseliflavus* [6].

*E. casseliflavus* is an unlikely pathogen but it can cause invasive infections. A review of the literature reveals numerous reports of *E. casseliflavus* bacteraemias mainly in patients with serious comorbidities, such as hematologic malignancy, receipt of organ transplant, renal failure, diabetes mellitus, and antithrombin III deficiency [6]. Cases of vascular and iv line-related infection have also been described [10,11].

Various studies have proven that *E. casseliflavus* colonizes the gastrointestinal tracts of both hospitalized and non-hospitalized healthy individuals, and no definite risk factors for colonization have been identified [1,6,12]. The previous use of vancomycin is not associated with the carriage of *E. casseliflavus* [1]. The source of *E. casseliflavus* is probably the food chain since it has been isolated from farm animals and vegetables [13,14].

Vancomycin therapy for enterococcal strains with MICs of 4 μg/mL or lower is regarded as adequate according to EUCAST. However, there have been cases in which the use of vancomycin for in vitro susceptible strains of *E. casseliflavus* has led to therapeutic failure or breakthrough bacteremia, therefore vancomycin is not recommended for the therapy of *E. casseliflavus* infection [2,6,15]. The correct species identification is crucial for the right choice of therapy. In our case, the patient was treated with a combination of daptomycin and gentamicin initially, and then ampicillin and ceftriaxone, according to the antibiotic susceptibility profile of the isolate.

Furthermore, it is important to identify correctly the enterococcal species that have low level vancomycin resistance and may be misidentified as *vanB*, in order to prevent costly unwarranted infection control measures. *E. casseliflavus* has not been associated with nosocomial outbreaks, and it is not an infection control problem [6,16,17].

*E. casseliflavus* is a rare pathogen but it has to be considered, especially in cases of infections in immunocompromised hosts, as early accurate diagnosis increases the rates of cure and survival.
